# The conundrum in diagnosing Maturity-Onset Diabetes of the Young (MODY) in a large German pedigree with early-onset diabetes and a novel *HNF1A* variant

**DOI:** 10.1186/s40348-026-00229-0

**Published:** 2026-04-10

**Authors:** Eleni Z. Giannopoulou, Abubakar Moawia, Josef Högel, Joanna Lerner, Stefanie Zorn, Christian Denzer, Reiner Siebert, Martin Wabitsch

**Affiliations:** 1 Division of Pediatric Endocrinology and Diabetes, Department of Pediatrics and Adolescent Medicine, University Medical Center Ulm, Ulm, Germany; 2 Center for Rare Endocrine Diseases at the University of Ulm, Ulm, Germany; 3 Institute of Human Genetics, Ulm University and Ulm University Medical Center, Ulm, Germany; 4 German Center for Child and Adolescent Health (DZKJ), Partner Site Ulm, Ulm, Germany

**Keywords:** MODY, Next generation sequencing, Sulfonylurea

## Abstract

**Background:**

Genetic screening for maturity-onset diabetes of the young (MODY) involves sequencing of the coding regions of known disease-associated genes. We describe the complex and challenging diagnostic journey of a patient with early-onset diabetes with a novel, intronic *HNF1A* variant likely affecting a branching site.

**Case presentation:**

The patient was diagnosed with diabetes at the age of 10 years after incidental hyperglycemia (HbA1c 8.1%, C-peptide 3.0 μg/dl), without polyuria, polydipsia, or weight loss. Type 1 diabetes associated autoantibodies were negative, but the patient had a strong family history of early-onset diabetes (classified as type 1 or type 2 diabetes). Initial genetic testing for *HNF4A*, *GCK*, *HNF1A*, and *HNF1B* coding regions (including exon/intron boundaries ± 20 bp) and MLPA were negative. Sulfonylureas provided good glycemic control until age 16, when insulin was added. At age 18, an expanded targeted next-generation sequencing (NGS) panel for MODY was also negative. At age 24, whole-exome-sequencing via NGS and additional analysis was conducted, focusing on synonymous and intronic variants, and revealed a heterozygous *HNF1A* variant (c.327-28A > G;p.?) in the patient and four affected relatives. The variant co-segregated with diabetes, and was predicted to affect splicing via branching site disruption, suggesting pathogenicity.

**Conclusion:**

In summary, this case highlights the importance of a comprehensive diagnostic approach that combines clinical, biochemical, and extended genetic evaluation. When MODY is strongly suspected despite negative targeted testing, broader sequencing—including intronic and regulatory regions—should be pursued. Accurate variant interpretation remains essential to prevent misclassification and to optimize diagnosis, treatment, and understanding of the genetic complexity of monogenic diabetes.

**Supplementary Information:**

The online version contains supplementary material available at 10.1186/s40348-026-00229-0.

## Introduction

Monogenic diabetes refers to a diverse group of disorders characterized by hyperglycemia, primarily caused by a pathogenic variant in a single gene. This alteration results in impaired function or abnormal development of the islets of Langerhans, particularly the insulin-secreting pancreatic β-cells. Monogenic diabetes encompasses neonatal diabetes, maturity-onset diabetes of the young (MODY) and some diabetes-associated syndromes [3]. Among these, MODY is the most prevalent form of monogenic diabetes, which may account for 0.2–5% of patients living with diabetes [3, 7, 15, 26]. The exact prevalence of MODY diabetes is likely significantly underestimated in many countries due to limited or unavailable genetic testing, insufficient awareness among healthcare providers, and the overlap of clinical features with more prevalent forms of diabetes, like type 1 and type 2 diabetes [3].

MODY is primarily defined by impaired insulin secretion with minimal or no defects in insulin action. MODY is typically characterized by early-onset mild hyperglycemia, classically before the age of 25 years, although diagnosis may also occur later in life [2]. Hyperglycemia may worsen over time depending on the involved gene and the nature of the underlying genetic variant [3]. MODY usually follows an autosomal dominant inheritance pattern, with pathogenic variants identified in one of at least 15 genes that variably impact beta-cell function [3, 5]. Recent studies, however, have called for the reassessment of several genes previously classified as causative of MODY, including *BLK* (formerly MODY11), *KLF11* (formerly MODY7), *PAX4* (formerly MODY9), *APPL1* (formerly MODY14), and *WFS1*, as emerging variant- and gene-level evidence indicates that alterations in these genes are unlikely to result in a MODY phenotype [16, 23].The most frequently reported subtypes remain GCK-MODY (formerly MODY2), HNF1A-MODY (formerly MODY3), and HNF4A-MODY (formerly MODY1) [2, 3, 5].

MODY serves as an excellent example of how precision medicine can transform diagnosis and treatment. By identifying the specific pathogenic variant, clinicians can tailor therapies to the individual's underlying genetic alteration, leading to more accurate diagnoses, targeted treatments, and improved patient outcomes. For instance, individuals with GCK-MODY typically present with mild fasting hyperglycemia that generally does not require antidiabetic treatment, except during pregnancy [3]. In contrast, individuals with HNF1A-MODY or HNF4A-MODY usually demonstrate a favorable response to low doses of sulfonylureas, while in certain cases, insulin therapy may become necessary in the course of the disease [2]. Furthermore, determining the molecular cause of diabetes in an individual enables early screening and proactive management of associated complications and offers crucial information regarding the risk of familial recurrence, aiding in genetic counseling and preventive care for at-risk relatives.

To fully unlock the diagnostic potential for individuals with monogenic diabetes, comprehensive genetic testing is essential [3, 27]. Here, we present the challenging diagnostic journey of a patient with early-onset diabetes mellitus and the presence of an intronic variant in *HNF1A* likely to affect branch point motif, that was missed by targeted testing of coding exons with a custom gene panel.

## Case presentation

**Fig. 1:**
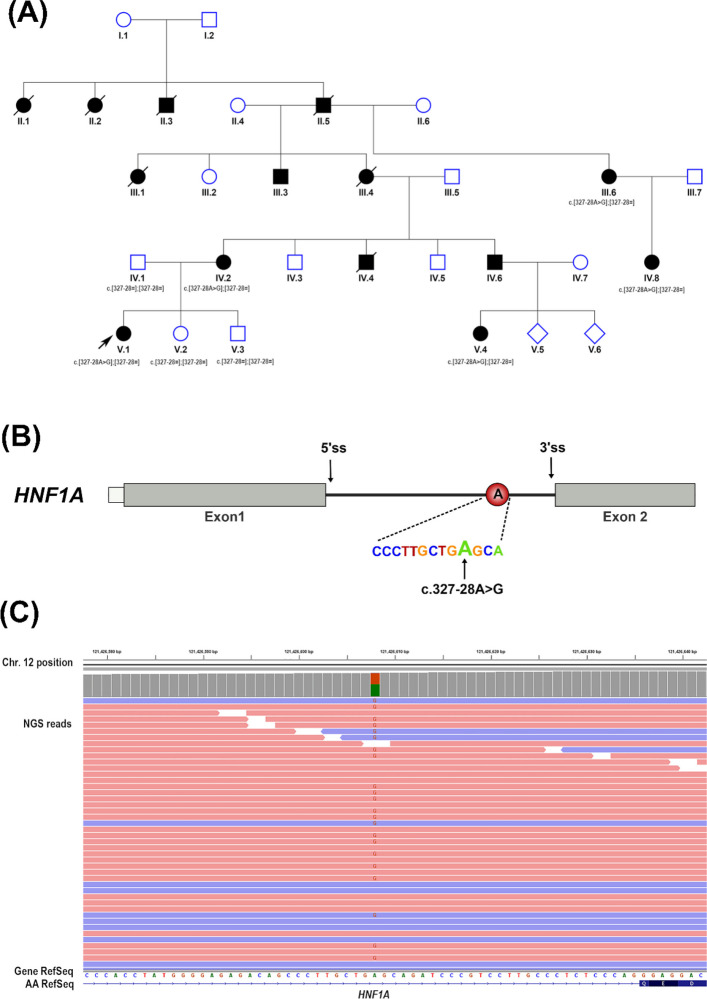
**A** Pedigree of the reported family with *HNF1A* MODY. Squares represent males, circles indicate females and diamonds show unknown sex. A shape with a diagonal line means deceased. Filled black shapes indicate members affected with early-onset diabetes, treated either with oral antidiabetic drugs or insulin. Proband is indicated by a black pointed arrow (ID V-1). Genotypes for each analyzed individual are displayed below the shapes following HGVS nomenclature (**B**) Schematic illustration of first and second exon of *HNF1A*. Exons are represented by grey boxes and the intron 1 by a black straight line between the exons. 5’ splice site (5’ss) and 3’ splice site (3’ss) are indicated by black arrows. MODY-associated variant in *HNF1A* is present near exon 2 splice acceptor site. The identified variant position is predicted to disrupt the function branch point motif (indicated in red circle) by numerous prediction tools. Sequence surrounding the *HNF1A* branch point variant are also shown (**C**) The variant in *HNF1A* identified by NGS. At the variant position, the total depth was 127 reads, of which 66 reads supported the reference and 61 reads the alternate allele (48%), consistent with the expected ratio for a heterozygous germline variant. The forward reads were colored pink and the reverse reads blue. All annotations, coordinates, and variant descriptions were based on GRCh37/hg19 assembly (NM_000545.8)

We present a family of German origin with multiple members affected by early-onset diabetes mellitus (shown in Fig. 1). Data from the reported family members were collected and analyzed retrospectively.

The index patient, a 10-year-old girl, was admitted to the Department of Pediatrics and Adolescent Medicine at the University Medical Center in Ulm, Germany for further evaluation following an incidental finding of hyperglycemia after a minor finger injury. The girl mentioned no symptoms of polyuria, polydipsia or weight loss, and no symptoms of infection. She had no known medical conditions and was not taking any medication. Her body weight was 42 kg (75th—90th percentile for age and sex [14]), her height was 144 cm (25th—50th percentile for age and sex [14]) and her body mass index (BMI) was 20.3 kg/m^2^ (75th – 90th percentile for age and sex [14]). Clinical examination revealed no pathologic findings, particularly no presence of acanthosis nigricans. Laboratory examination revealed an elevated HbA1c of 8.1%, fasting C-peptide was 3.0 μg/dl, fasting insulin was 12.7 mU/l and type 1 diabetes associated autoantibodies (glutamic acid decarboxylase antibodies [GADA], islet antigen 2 antibodies [IA-2A], insulin autoantibodies [IAA] and zinc transporter 8 [ZnT8] antibodies) were negative. An oral glucose tolerance test (OGTT) confirmed diabetes mellitus (fasting plasma glucose 155 mg/dl, 30-min plasma glucose 267 mg/dl, 60-min plasma glucose 343 mg/dl, 90-min plasma glucose 353 mg/dl, 120-min plasma glucose 376 mg/dl), with relatively low insulin levels throughout, consistent with relative insulin deficiency (fasting insulin 11.8 pmol/l, 30-min insulin 120.8 pmol/l, 60-min insulin 104.0 pmol/l, 90-min insulin 101.4 pmol/l, 120-min insulin 88.2 pmol/l) [12]. Lipid levels, including total cholesterol, LDL-cholesterol, HDL-cholesterol and triglycerides were within the normal range.

The patient had a significant family history of early-onset diabetes (shown in Fig. 1). Her mother was diagnosed with diabetes at the age of 15 years and, due to clinical suspicion of MODY diabetes, she was initially treated with sulfonylurea. However, as her glycemic control progressively worsened over time, it became necessary to transition her at the age of 31 years to insulin therapy in order to optimize glucose regulation. The mother of the index patient is currently on insulin pump therapy. While she had a normal body weight in early adulthood, she is now obese (BMI: 34.3 kg/m^2^). The patient’s maternal grandmother had a history of kidney cysts and developed diabetes at the age of 30 years, for which she was treated with oral antidiabetic agents. Furthermore, multiple maternal relatives were diagnosed with early-onset diabetes and were treated with either oral antidiabetic drugs (sulfonylurea or metformin) or insulin. However, none of the affected family members had a confirmed diagnosis specifying the subtype of their diabetes; the majority were presumed to have type 2 diabetes mellitus due to overweight or obesity.

Given the patient’s clinical presentation and family history, monogenic diabetes was suspected. Genetic sequencing of the coding regions of the genes associated with HNF4A-MODY, GCK-MODY, HNF1A-MODY and HNF1B-MODY (incl. exon/intron boarders ± 20 base pairs (bp) beyond the exon boarder), as well as multiplex ligation-dependent probe amplification (MLPA) analysis, was conducted at the time of diagnosis for the index patient. The analyses did not identify any pathogenic variants, deletions or duplications in the examined genes (*HNF4A*, *GCK*, *HNF1A*, *HNF1B*); however, due to strong suspicion of MODY diabetes, treatment with sulfonylureas was started.

During sulfonylurea treatment, frequent dose adjustments were required, gradually increasing to a maximum of 6 mg/day over a period of 6 years after diagnosis. With these dose adjustments, good glycemic control was maintained, with HbA1c levels remaining consistently below 7.0%. During this period, two additional family members, a maternal uncle and a maternal cousin, were identified with elevated blood glucose levels during presymptomatic screening examinations (shown in Fig. 1). At the age of 16 years, the index patient was started on insulin therapy due to rising HbA1c levels (up to 8.7%). The patient was initially started on prandial insulin, and after one year, basal insulin supplementation was introduced. However, basal insulin was discontinued shortly after initiation due to recurrent hypoglycemia at night. Since then, the patient is treated with prandial insulin injections. Despite transition to insulin, the patient continued to have detectable C-peptide levels and negative type 1 diabetes autoantibodies.

For several years, despite persistent suspicion, no molecular diagnosis could be established. At the age of 18 years, a further genetic testing including next-generation sequencing (NGS) of the coding regions of a panel of genes related to MODY types 6–14 (*NEUROD1*, *KLF11*, *CEL*, *PAX4*, *INS*, *BLK*, *ABCC8*, *KCNJ11*, *APPL1*) was performed, but yielded negative results. In addition, MLPA analysis of the *NEUROD1*, *KLF11*, *CEL*, *PAX4* and *INS* gene regions did not identify any deletions or duplications. Finally, at the age of 24 years, whole-exome-sequencing via NGS (TWIST™ exome kit, NextSeq High Output Kit v2.5) and additional analysis was conducted, focusing on synonymous and (deep-)intronic variants. A hitherto not reported heterozygous variant exchanging one nucleotide in the intron 1 of *HNF1A* (c.327-28A > G; p?, NM_000545.8) was identified in the proband. Though formal linkage analysis was underpowered, the variant showed perfect segregation with the disease within the family (shown in Fig. 1a,c). The mother of the index patient, as well as two maternal cousins and a maternal aunt, all with a history of mild hyperglycemia or diabetes mellitus, agreed to undergo genetic testing and were found positive for this variant (shown in Fig. 1a). The father (healthy, BMI 25.2 kg/m^2^) and the two siblings of the index patient (BMI of the brother: 25.7 kg/m^2^, BMI of the sister: 33 kg/m^2^, both without hyperglycemia or diabetes mellitus) were tested negative for this variant (shown in Fig. 1a).

This variant has never been reported in ClinVar database, Leiden Open Variation Database (LOVD) database, HGMD® Professional v2025.2, or gnomAD v4.1.1 database (last accessed August 01, 2025) and in the literature. The variant resides in a partially conserved intronic element according to PhyloP100way scores: 2.9/1 [deleterious, phyloP > 2.0 [8]] and was predicted to effect a branch point (BP) motif (Fig. 1b). BP variants may compromise the assembly of the spliceosome, leading to changes in proper splicing and gene expression. We submitted the sequence in the region affected by the variant to splice site prediction programs (La Branchor (http://bejerano.stanford.edu/labranchor), BranchPoint Hunter (https://hgidsoft.rockefeller.edu/BPHunter/),S-CAPv1.0 (http://bejerano.stanford.edu/scap/),SpliceAPP (https://bc.imb.sinica.edu.tw/SpliceAPP/) and Splice AI (https://spliceailookup.broadinstitute.org/). All these tools corroborated that the variant affects splicing, except for Splice AI. Full splice prediction scores for the variant are shown in Supplementary Table 1. According to the ACMG/AMP variant classification criteria, the *HNF1A*:c.327-28A > G; p? variant could be classified as ‘variant of unknown significance’ (PP4-Supporting, PM2-Moderate, PP1- Supporting) [21]. RNA sequencing to further assess the pathogenicity of the variant concerning splicing was not feasible due to the low expression of *HNF1A* in accessible patient-derived materials, including blood, as indicated by the GTEx database (https://gtexportal.org/home/gene/HNF1A). No other pathogenic or likely pathogenic variant in the *HNF1A* gene region covered by the TWIST exome sequencing was detected.

Considering the clinical presentation, biochemical profile, response to sulfonylurea therapy, family history, segregation data, and in silico predictions, the identified intronic *HNF1A* variant represented the most plausible, albeit not definitive, molecular explanation for the diabetes phenotype in this family.

## Discussion

The present report describes the complex and prolonged diagnostic journey of a patient with early-onset diabetes mellitus of uncertain etiology. Initially missed by targeted screening of coding regions, a novel, intronic *HNF1A* variant (c.327-28A > G; p?) of unknown significance was identified after comprehensive genetic analysis included intronic regions. The decision to classify it as the likely cause of HNF1A-MODY was driven by the cumulative evidence of its complete co-segregation with the disease, phenotypic features and sulfonylurea response, and supporting in silico predictions of a splicing defect. Although SpliceAI did not predict a pathogenic splicing effect for the identified *HNF1A* variant c.327‑28A > G, several branchpoint‑focused tools (LaBranchoR, BPHunter and S‑CAP) consistently indicated disruption of the intronic branchpoint motif. Notably, the identified *HNF1A* variant is predicted to affect the adenine within the branchpoint sequence (“branchpoint A”) located 28 nucleotides upstream of the intron 1 3′ splice site, thereby disrupting this branchpoint. According to the literature, the branchpoint is a single‑nucleotide *cis*‑acting element within the intron, typically an adenine, and is generally located approximately 20–40 nucleotides upstream of the 3′ splice site [11]. Taken together, these findings support the classification of this variant as a plausible branchpoint-disrupting change, and justifying functional follow-up.

This case underscores the significant challenges in achieving a definitive genetic diagnosis for unsolved MODY cases and emphasizes the critical need to expand testing beyond exonic regions when clinical suspicion is high. *HNF1A*-MODY (formerly MODY3) is one of the most common MODY subtypes and is characterized by progressive hyperglycemia that leads to diabetes with a high risk of developing chronic vascular complications [5, 28]. Early initiation of appropriate therapy can reduce the risk of complications in patients with this disorder. Patients with HNF1A-MODY typically respond well to oral hypoglycemic agents, particularly sulfonylureas; however, insulin therapy may become necessary as the disease advances [3, 5]. Recent clinical evidence suggests that incretin mimetic drugs, like GLP-1 receptor agonists, demonstrate efficacy in patients with HNF1A-MODY who respond poorly to sulfonylurea therapy [4]. Still, in the absence of a genetic diagnosis, patients with HNF1A-MODY are often misdiagnosed and treated with insulin injections.

Targeted genetic screening for MODY typically involves sequencing of known disease-associated genes [3]. Intronic splice altering variants outside of the consensus splice site region, as described here, are very likely to be underreported amongst MODY-causing genes due to a lack of detection by targeted genetic testing focusing on coding regions [6, 27]. For patients who present with a strong clinical suspicion of MODY but have negative results on targeted testing, broader approaches—such as whole-gene, exome, or genome sequencing—should be considered to uncover rare or novel variants in noncoding regions of known MODY genes or in yet undiscovered MODY loci [9].

Beyond technical limitations, variant interpretation itself poses substantial challenges. The increasing availability of sequencing platforms has expanded the catalog of *HNF1A* variants in the HGMD® Professional v2025.2—over 800 reported to date—yet not all exert a clear pathogenic effect (http://www.hgmd.cf.ac.uk/ac/index.php). Some rare *HNF1A* variants act as modest type 2 diabetes risk alleles rather than monogenic causes, while others may be benign [1, 16, 20, 23]. Such findings may result in misdiagnosis, potentially causing unnecessary anxiety and additional testing in families. Rigorous classification following ACMG guidelines, integrating population data, segregation studies, clinical phenotype, and functional assays, remains indispensable for accurate interpretation.

According to the American Diabetes Association, MODY should be suspected in patients lacking typical features of type 1 or type 2 diabetes—specifically those with negative autoimmune markers, absence of obesity, and a strong family history of diabetes [2]. However, distinguishing MODY from atypical forms of diabetes remains challenging, as occasional autoantibody positivity, de novo mutations, and the rising prevalence of childhood obesity can obscure diagnosis [13, 15, 24, 25]. To aid clinical assessment, the MODY probability calculator has been developed using key clinical variables such as age at onset, treatment, BMI, and family history. Although it performs well in differentiating MODY from type 1 diabetes, its accuracy is lower for distinguishing MODY from type 2 diabetes—especially in youth populations—but it remains a valuable tool for guiding genetic testing and improving diagnostic precision [18, 22].

In the present study clinical, laboratory, and segregation data were highly consistent with a MODY diagnosis in the index patient. Furthermore, a sustained therapeutic response to sulfonylureas for over six years following diagnosis provided additional functional evidence supporting HNF1A-MODY, although such response is not formally included in ACMG criteria [21]. Type 2 diabetes was considered improbable due to the patient’s young age, absence of obesity or metabolic comorbidities like dyslipidemia and insulin resistance, and healthy lifestyle [2]. Although the identified *HNF1A* intronic variant was not classified as pathogenic, the phenotype co-segregated with diabetes in affected relatives and was absent in unaffected family members. Collectively, these findings strongly support the variant’s likely pathogenic role in HNF1A-MODY.

An important observation from our study was the marked phenotypic variability in disease severity among individuals carrying the same *HNF1A* variant. The index patient and her mother exhibited similar phenotypes, characterized by early-onset diabetes requiring pharmacological treatment. In contrast, three additional family members—who were not first-degree relatives with the index patient—tested positive for the same variant but showed only mild hyperglycemia that did not require medical therapy. These individuals underwent testing following the identification of the variant in the index case. Unfortunately, genetic testing could not be performed in other relatives with more severe diabetes, as some were either deceased or declined participation.

Consistent with our findings, previous studies have reported considerable phenotypic variation among heterozygous carriers of rare, pathogenic *HNF1A* variants [12, 19]. According to their results, this heterogeneity is likely influenced by each individual’s polygenic background, particularly the presence of common type 2 diabetes risk alleles, that can significantly modify both the penetrance and clinical expression of pathogenic mutations [12, 19]. Notably, carriers of *HNF1A* variants with a low type 2 diabetes polygenic risk score may have a substantially reduced likelihood of developing diabetes, with up to half remaining disease-free in population-based studies [19]. Collectively, these findings underscore that the pathogenesis of MODY involves complex polygenic interactions rather than a purely deterministic monogenic mechanism, highlighting the need to reconsider diagnostic and risk assessment frameworks for monogenic diabetes [19]. In our study, genome-wide data were not available, so the potential contribution of common type 2 diabetes–associated variants to disease expression could not be assessed. Further comprehensive genomic analysis is warranted to explore the role of such “hidden” variants in modulating the phenotype.

An important limitation of the present study is that no functional studies were performed in order to further investigate the pathogenicity of the variant, as suggested by the ACMG guidelines for variant interpretation [17, 21]. Especially for *HNF1A* variants it has been shown that while some *HNF1A* variants can cause HNF1A-MODY, other variants can be risk factors for the development of type 2 diabetes or even be benign [1, 20]. As discussed above, functional data can provide important supportive evidence for variant classification but should not be considered in isolation when assessing pathogenicity. The same variant may lead to variable clinical manifestations depending on the influence of additional genetic modifiers and environmental factors, which functional assays alone cannot fully capture [1, 10]. Another limitation of this study is that genetic testing was not performed in further family members with a severe diabetes phenotype, as they were either deceased or declined participation. Testing these individuals would have provided additional evidence supporting the pathogenicity of the identified variant.

## Conclusion

In summary, this case underscores the need for a comprehensive diagnostic framework that integrates clinical assessment, biochemical profiling, and extended genetic analysis. In patients with high clinical suspicion of MODY but negative targeted testing, reanalysis using broader sequencing approaches—including intronic and regulatory regions—should be strongly considered. Equally important, careful variant interpretation and, where possible, functional validation are critical to avoid over- or underestimation of pathogenicity. Ultimately, improving diagnostic accuracy for MODY will not only refine patient management and therapy selection but also enhance our understanding of the genetic and polygenic architecture underlying monogenic diabetes.

## Supplementary Information

Supplementary Material 1.

## Data Availability

The dataset(s) supporting the conclusions of this article is(are) included within the article (and its additional file(s). Further enquiries can be directed to the corresponding author.

## Abbreviations

MODY: Maturity-onset diabetes of the young
BMI: Body mass index
GADA: Glutamic acid decarboxylase antibodies
IA-2A: Islet antigen 2 antibodies
IAA: Insulin autoantibodies
ZnT8: Zinc transporter 8
MLPA: Multiplex ligation-dependent probe amplification
NGS: Next-generation sequencing
