# Supplementary material for: The conundrum in diagnosing Maturity-Onset Diabetes of the Young (MODY) in a large German pedigree with early-onset diabetes and a novel HNF1A variant
Source: Mol Cell Pediatr. 2026 Apr 10;13:16. doi: 10.1186/s40348-026-00229-0 (PMC13069046; doi:10.1186/s40348-026-00229-0)
Supplement: Supplementary file 1 — Supplementary Material 1. [file 40348_2026_229_MOESM1_ESM.docx]

| Prediction tool  (score range) | Score | Interpretation |
| --- | --- | --- |
| BranchPoint Hunter  (0–1) | **0.60**  (cutoff **score > 0.5**) | Alteration of the branchpoint, most probably affecting splicing |
| La Branchor  (0–1) | **0.75**  (cutoff **score ≥ 0.66**) | variant impact to the splice branch point |
| S-CAP | **0.034**  (cutoff **score for**  **3' intronic variants > 0.006**) | predicted to be potentially splice-disruptive |
| SpliceAPP | **0.2846**  (cutoff **score** 3' splice site (3'SS) non-AG **> ≥ 0.0778)** | Variant may impact normal splicing |
| SpliceAI  (0–1) | 0.030  (cutoff **score> 0.2)** | Benign |

Supplementary table 1. *In silico* analysis of the effect of the *HNF1A* c.327-28A>G (NM_000545.8) variant on splicing (last accessed August 01, 2025).
